# Quality Evaluation of *Tricholoma matsutake* Based on the Nucleic Acid Compounds by UPLC-TOF/MS and UPLC-QqQ/MS

**DOI:** 10.3390/molecules24010034

**Published:** 2018-12-21

**Authors:** Ying Xue, Wei Jin, Xian-Shun Xu, Li Yong, Bin Hu, Jing Xiong, Xue-Mei Hu, Lin-Sen Qing, Jing Xie

**Affiliations:** 1 School of Pharmacy, Collaborative Innovation Center of Sichuan for Elderly Care and Health, Chengdu Medical College, Chengdu 610500, China; xuecher0221@sina.com (Y.X.); s_scu_xiongjing@163.com (J.X.); xuemei.hu@163.com (X.-M.H.); 2 Chengdu Institute of Biology, Chinese Academy of Sciences, Chengdu 610041, China; 3 Sichuan Provincial Center for Disease Control and Prevention, Chengdu 610041, China; qgmw6970@163.com (W.J.); xuxianshun@163.com (X.-S.X.); yongch121@163.com (L.Y.); hubin1258@163.com (B.H.)

**Keywords:** *Tricholoma matsutake*, quality evaluation, nucleic acid compound, UPLC-TOF/MS, UPLC-QqQ/MS

## Abstract

So far, there has been no quality evaluation of *Tricholoma matsutake*. Nucleic acid compounds are a kind of functional ingredient in *T. matsutake* that is beneficial to human health. In this study, a UPLC-TOF/MS method was first used to scan and identify the potential nucleic acid compounds in *T. matsutake*. Based on the calculation of the molecular formula and subsequent confirmation by authentic standards, 15 nucleic acid compounds were unambiguously identified: adenosine, cytidine, guanosine, inosine, thymidine, uridine, xanthosine dehydrate, 2′-deoxyadenosine, 2′-deoxycytidine, 2′-deoxyguanosine, 2′-deoxyuridine, adenosine 5′-monophosphate, cytidine 5′-monophosphate, guanosine 5′-monophosphate, and uridine 5′-monophosphate. Then, a UPLC-QqQ/MS method was developed for the subsequent quantitative analysis. After validating the limits of quantification, detection, precision, repeatability, and recovery through a calibration curve, the content of 15 nucleic acid compounds was determined by the proposed UPLC-QqQ/MS method in 80 *T. matsutake* samples collected from different regions in Sichuan province, Southwest China. After the statistical analysis, we suggest that the total content of nucleic acid compounds in the qualified *T. matsutake* should be higher than 24.49 mg/100 g. The results indicated that the combined use of UPLC-TOF/MS and UPLC-QqQ/MS is efficient for fast identification and determination of nucleic acid compounds to comprehensively evaluate the quality of *T. matsutake*.

## 1. Introduction

*Tricholoma matsutake*, a natural, wild, edible fungus, is an endemic species in East Asia. The Hengduan Mountain range region of Southwest China, especially the Sichuan province, is the world’s main production area of *T. matsutake*. China, Europe, and Japan are the world’s three major consumer markets. *Tricholoma matsutake* is widely used in high-end food because of its unique flavor and fresh taste. Additionally, it is also made into health products with diverse biological activities, such as antioxidant [1,2], immuno-modulating [3,4], and anti-tumor [5,6] activity. However, so far, there has been no quality evaluation of *T. matsutake*.

Ying et al. reported 5 nucleic acid constituents of *T. matsutake* for the first and only time [7]. Nucleic acid components are the most important functional small molecule ingredients in edible fungi. Nucleic acid components are made of DNA and RNA precursor molecules and play crucial roles in almost all cellular functions. They regulate various physiological processes through the purine/pyrimidine receptors in the human body [8,9]. Eating foods rich in nucleic acid compounds can help in the recovery from diseases and enhance the immune system. Therefore, nucleic acid constituents are suitable and quantifiable markers for the evaluation of the quality of *T. matsutake*.

Sichuan Food and Drug Administration commissioned our team and other researchers to jointly draft the local standards for the safety of *T. matsutake* and its products, which were released and implemented on 20 July 2018 [10]. These local standards only stipulate food safety requirements such as pesticide residues, heavy metal residues, and microbial limits, etc. Time-of-flight mass spectrometry is commonly used in the discovery of unknown compounds in a complex matrix [11]. When target compounds are located and identified, QqQ/MS is an excellent choice for the subsequent quantitative analysis [12]. In this study, 15 nucleic acid compounds in 80 *T. matsutake* samples from different regions from Sichuan province were firstly screened by UPLC-TOF/MS and subsequently determined by UPLC-QqQ/MS to comprehensively evaluate the quality of *T. matsutake*.

## 2. Results and Discussion

### 2.1. Scanning and Identification of Nucleic Acid Compounds by UPLC-TOF/MS

Since only one research article has preliminarily reported the existence of 5 nucleic acid compounds in *T. matsutake* [7], UPLC-TOF/MS was first used to scan and identify the potential nucleic acid compounds in *T. matsutake*. TOF/MS is often used for qualitative analysis, since it is able to determine the high-resolution molecular mass of target compounds, enabling qualitative structure identification in a complex matrix [13]. When target compounds are located and identified, QqQ/MS is an excellent choice for the subsequent quantitative analysis [14].

**Figure 1:**
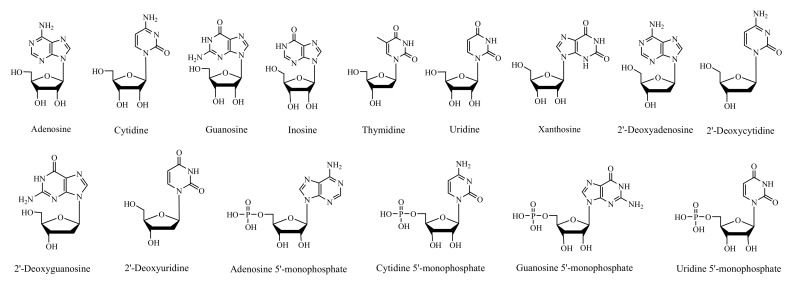
Chemical structures of 15 nucleic acid constituents.

In this study, based on the calculation of the molecular formula and subsequent confirmation by authentic standards under the same chromatographic conditions, 15 nucleic acid compounds (Figure 1) were unambiguously identified in *T. matsutake*: adenosine (A), cytidine (C), guanosine (G), inosine (I), thymidine (T), uridine (U), xanthosine dehydrate (X), 2′-deoxyadenosine (dA), 2′-deoxycytidine (dC), 2′-deoxyguanosine (dG), 2′-deoxyuridine (dU), adenosine 5′-monophosphate (AMP), cytidine 5′-monophosphate (CMP), guanosine 5′-monophosphate (GMP), and uridine 5′-monophosphate (UMP). As shown in Table 1, the error of each compound in high-resolution mass spectral data was within ±5 ppm, which is an acceptable error limit for structure confirmation [15,16].

### 2.2. Determination of Nucleic Acid Compounds by UPLC-QqQ/MS

#### 2.2.1. Optimization of the UPLC-QqQ/MS Condition

**Figure 2:**
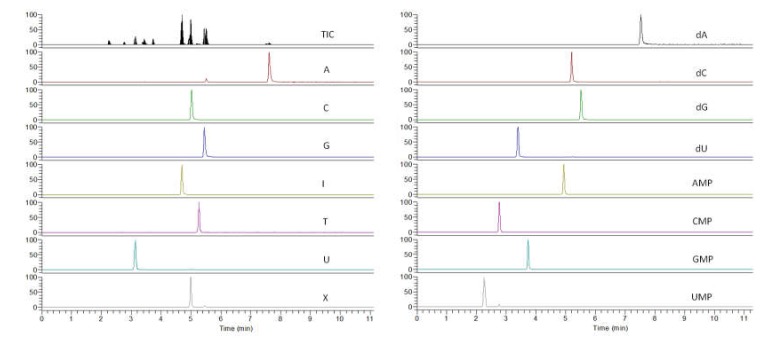
Ultra-performance liquid chromatography mass spectrometer chromatograms of 15 nucleic acid constituents.

After optimizing the types of analytical column, the solvent composition, and the pH of the mobile phase and the MS parameters, the UPLC-QqQ/MS conditions for the quantitative analysis of nucleic acid compounds were determined [14,17]. The typical mass spectra are shown in Figure 2.

#### 2.2.2. Analytical Figures of Merit

Method validation was implemented under the above UPLC-QqQ/MS conditions. The calibration curves of 15 target analytes were firstly determined by the mixed working solutions I-V. Binary linear regression analysis was performed as shown in Table 2 by taking the concentration of each authentic standard as the abscissa (*x*) and the corresponding peak area as the ordinate (*y*), respectively. The limit of quantification (LOQ) and the limit of detection (LOD) were measured by a gradual dilution process of the standard stock solutions until the signal-to-noise ratio (S/N) was 10:1 and 3:1, respectively. The precision was evaluated by the relative standard deviation (RSD) using standard working solution III, which was tested within one day (0 h, 4 h, 8 h, 12 h, 16 h, and 24 h) to determine the intra-day precision, and was tested within 3 days to determine the inter-day precision. The repeatability was evaluated by analyzing six independent portions of sample S-05. The recovery was evaluated by spiking an amount of about 1:1 of authentic standards to sample S-05 in parallel operation six times. The results summarized in Table 2 show that the analytical method meets the requirements for a quantitative analysis and was appropriate for the determination of 15 nucleic acid constituents in *T. matsutake*. Besides, the key values of method validation in this work were compared with those of several other quantitative methods reported, as shown in Table S1.

### 2.3. Quality Evaluation of T. matsutake Samples

Eighty batches of *T. matsutake* were collected by CDC staff from Sichuan province. The contents of 15 nucleic acid compounds were determined by the proposed UPLC-QqQ/MS method. The results are shown in Table 3.

The nucleic acid compounds in this study could be divided into three categories: nucleosides, deoxynucleosides, and mononuclear nucleotides. In general, the total content of nucleosides was the highest, followed by mononuclear nucleotides, and the content of deoxynucleosides was the lowest. Four nucleosides (T, X, dU, and dC) could not be detected in some samples. The mononuclear nucleotides are typical flavoring nucleotides and can significantly enhance the umami taste together with umami amino acid (aspartic acid and/or glutamic acid) [18].

**Figure 3:**
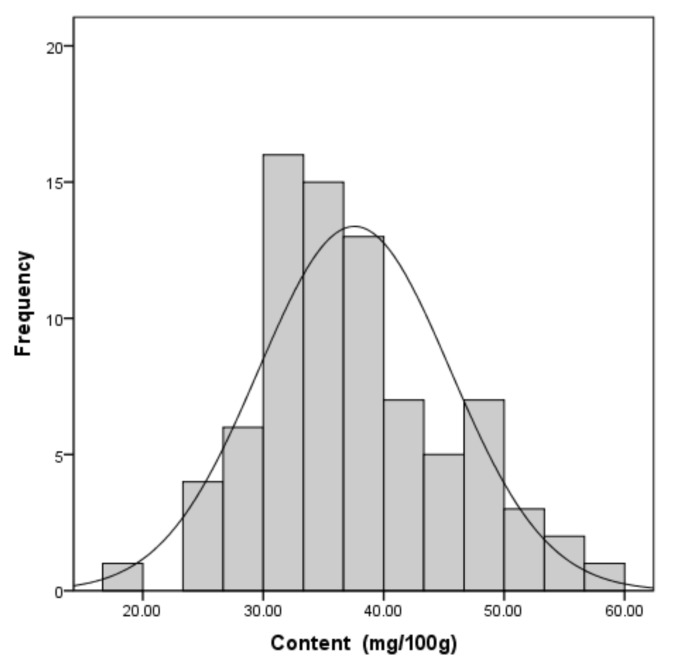
The stem-and-leaf plot of normal distribution plot.

The total content of nucleic acid compounds was 37.57 ± 7.95 mg/100 g (μ ± σ, average value ± standard deviation), which suggests that there was little difference in nucleic acid content among samples from different regions. The data of the total content of nucleic acid compounds in 80 samples were further treated with the SPSS 19.0 statistical package. A descriptive statistical analysis was used according to the type of the data. To test significance, the t-test and variance analysis were used. The data of the total content of nucleic acid compounds conformed to a normal distribution since the results of the test of normality were *p* = 0.200 (>0.05), skewness = 0.463 (<1), and kurtosis = 0.23 (<1). The stem-and-leaf plot is shown in Figure 3. The reference range of the total content of nucleic acid compounds could be defined by normal distribution method. The low limit of the total content was calculated as 24.49 mg/100 g (μ − 1.645 σ, at the 0.05 level, one-tailed). Among the results of the 80 samples used in this work, the actual distribution (exceeding 24.49 mg/100 g) was 97.5%, which was greater than 95% of the theoretical distribution. In conclusion, we suggest that the total content of nucleic acid compounds in *T. matsutake* should be higher than 24.49 mg/100 g to be a qualified product.

## 3. Material and Methods

### 3.1. Chemicals and Reagents

Eighty dried samples of *T. matsutake* were collected in accordance with official sampling requirements [19] from the mountain areas of Xiaojin County, Jiulong County, Yajiang County, Kangding County, Muli County, and Lixian County in Sichuan Province, China. Fifteen authentic standards of adenosine (A), cytidine (C), guanosine (G), inosine (I), thymidine (T), uridine (U), xanthosine dehydrate (X), 2′-deoxyadenosine (dA), 2′-deoxycytidine (dC), 2′-deoxyguanosine (dG), 2′-deoxyuridine (dU), adenosine 5′-monophosphate (AMP), cytidine 5′-monophosphate (CMP), guanosine 5′-monophosphate (GMP), and uridine 5′-monophosphate (UMP) were obtained from Sigma-Aldrich (St. Louis, MO, USA). The Milli-Q water purification system was used to prepare ultra-pure water for the UPLC analysis (Millipore, Bedford, MA, USA). The solvent ammonium acetate, acetonitrile, and diethylamine with LC-MS grade for UPLC-MS analysis were also purchased from Sigma-Aldrich (Sigma-Aldrich, St.Louis, MO, USA). Other chemicals and solvents of analytical grade were purchased from Sinopharm Chemical Reagent Co., Ltd. (Shanghai, China).

### 3.2. Preparation of Standard Solution

Stock solutions of 15 target analytes were prepared by dissolving each 10 mg authentic standard in 100 mL ultra-pure water. Then, 1 mL of each of the 15 stock solutions was transferred to a 100 mL volumetric flask and diluted with ultra-pure water to obtain the working solution I of mixed standards with a concentration of approximately 10 μg/mL. Mixed working solutions II-V were obtained by diluting working solution I with respective concentrations of about 7.5 μg/mL, 5 μg/mL, 2.5 μg/mL, and 0.1 μg/mL. All solutions were stored at 4 °C before use.

### 3.3. Sample Preparation

One gram of *T. matsutake* powder was accurately weighed into a 100 mL volumetric flask, about 90 mL ultra-pure water was added, and this was kept in a boiling water bath for 15 min. Then, this was cooled to room temperature, the volume was fixed by ultra-pure water, and the extract of the *T. matsutake* sample was obtained. Finally, the solutions were filtered through a 0.22 μm syringe filter into HPLC vials for UPLC analysis.

### 3.4. Scanning and Identification of Nucleic Acid Compounds by UPLC-TOF/MS

The Shimadzu UPLC system (Shimadzu, Kyoto, Japan) consists of pumps (LC-30AD), an online degasser (DGU-20A^5R^), an auto-sampler (SIL-30AC), and a column oven (CTO-30aHE). Chromatographic separation was performed on a Thermo Hypercarb analytical column (2.1 mm × 100 mm, 5 μm) at 40 °C. The mobile phase consisted of an aqueous phase (2 mmol/L ammonium acetate solution containing 0.06% diethylamine, pH 11) and an organic phase (acetonitrile containing 2 mmol/L ammonium acetate and 0.06% diethylamine). The linear gradient elution was 2%~15%~40%~60% organic phase at 0~4~7.5~10 min. The constant flow rate was 0.6 mL/min. The sample solution of 2 µL was injected into the UPLC system by the auto-sampler.

Time-of-flight mass spectrometry measurements were performed on a 4600 Q-TOF mass spectrometer (AB Sciex, Concord, CA, USA) equipped with an electrospray ionization source. The Q-TOF/MS analysis was performed in negative mode with the following parameters: Ion source gas 1 (GS1) (N_2_) at 50 psi, ion source gas 1 (GS1) at 50 psi, curtain gas at 35 psi, a temperature of 500 °C, and an ionspray voltage floating at −4500 V. The mass range was set to *m*/*z* 100–1000. The system was operated under Analyst 1.6 and Peak 2.0 (AB Sciex, Concord, CA, USA) and used an APCI negative calibration solution to calibrate the instrument’s mass accuracy in real-time.

### 3.5. Determination of Nucleic Acid Compounds by UPLC-QqQ/MS

Chromatographic separation was performed under the same conditions as those used in the UPLC-TOF/MS analysis described above. The QqQ/MS measurements were accomplished by a triple quadrupole mass spectrometer equipped with an electrospray ionization source (ThermoFischer, TSQ VANTAGE, Waltham, MA, USA). The MS spectra were acquired in negative ion mode. The quantitative analysis of the target analytes was performed under multi-reaction monitoring (MRM) mode. The parameters of the mass spectrometry detector (MSD) were as follows: A vaporizer temperature of 350 °C, a capillary temperature of 350 °C, an aux gas pressure of 10 Arb, a sheath gas pressure of 40 Arb, an ion sweep gas pressure of 2 Arb, a discharge current of 4.0 µA, and a spray voltage of −2000 V. Data collection and processing were conducted with Thermo Xcalibur Workstation (Version 2.2, ThermoFischer, TSQ VANTAGE, Waltham, MA, USA).

## 4. Conclusions

In the present study, the combined use of UPLC-TOF/MS and UPLC-QqQ/MS was applied for the identification and determination of nucleic acid compounds in *T. matsutake*, including adenosine, cytidine, guanosine, inosine, thymidine, uridine, xanthosine dehydrate, 2′-deoxyadenosine, 2′-deoxycytidine, 2′-deoxyguanosine, 2′-deoxyuridine, adenosine 5′-monophosphate, cytidine 5′-monophosphate, guanosine 5′-monophosphate, and uridine 5′-monophosphate. The content of each nucleic acid compound was determined by the proposed UPLC-MS method in 80 *T. matsutake* samples collected from different regions in Sichuan province, Southwest China. The results show, for the first time, that *T. matsutake* is rich in nucleic acid compounds, and there is little difference in the nucleic acid content of *T. matsutake* from different locations in the Sichuan region. This shows that nucleic acid compounds should be incorporated into the quality standards as markers of *T. matsutake* and its products in the future.

## Figures and Tables

**Table 1:** Mass spectrometric parameters of 15 nucleic acid constituents in the negative ion-scan mode.

Analyte	TOF/MS		QqQ/MS	
	Quasi-Molecular Ion (*m*/*z*)	Error (ppm)	Parent Ion (*m*/*z*)	Product Ion (*m*/*z*)
A	266.09019	2.7	266	134 *, 107 ^#^
C	242.07717	−4.4	242	152 ^#^, 109 *
G	282.08436	−0.1	282	133 ^#^, 150 *
I	267.07231	−4.4	267	135 *, 92 ^#^
T	241.08235	−2.7	241	151 *, 125 ^#^
U	243.06279	2.2	243	152 ^#^, 110 *
X	283.06741	−3.5	283	151 *, 108 ^#^
dA	250.09446	−0.4	250	160 ^#^, 134 *
dC	226.08297	−1.6	226	135 ^#^, 93 *
dG	266.08899	−1.8	266	150 *, 133 ^#^
dU	227.06625	−4.8	227	184 *, 94 ^#^
AMP	346.05423	−4.6	346	211 ^#^, 79 *
CMP	322.04404	−1.7	322	211 ^#^, 79 *
GMP	362.0499	−2.3	362	211 ^#^, 79 *
UMP	323.02825	−1.1	323	211 ^#^, 79 *

Note: * quantitative ion, ^#^ qualitative ion.

**Table 2:** The results of method validation.

Analyte	Regression Equation	Linear Range (ng/L)	LOD	LOQ	Precision (RSD, *n* = 6)		Repeatability (*n* = 6)		Recovery (*n* = 6)	
	(y = ax + b, r^2^)		(ng)	(ng)	Intra-Day (%)	Inter-Day (%)	Mean (mg/100g)	RSD (%)	Mean (%)	RSD (%)
A	y = 240.695 + 6301.38x, 0.9998	0.12~11.15	0.04	0.12	2.71	3.99	12.29	0.77	99.1	2.33
C	y = 264.791 + 9060.44x, 0.9997	0.10~9.95	0.01	0.03	2.68	2.95	3.48	1.35	97.2	3.01
G	y = 1363.37 + 47042.3x, 0.9999	0.10~9.87	0.01	0.03	2.85	3.32	11.68	0.83	98.5	2.70
I	y = 5409.88 + 74348.5x, 0.9998	0.12~11.68	0.01	0.03	2.51	3.62	1.54	2.27	98.9	2.67
T	y = 166.893 + 2164.58x, 0.9992	0.10~9.92	0.03	0.09	1.38	2.60	0.20	3.01	96.3	4.38
U	y = −591.107 + 18137.6x, 0.9993	0.09~8.91	0.03	0.09	2.93	3.33	7.30	1.01	98.6	2.39
X	y = 3103.68 + 57804.6x, 0.9998	0.10~9.83	0.01	0.03	1.82	2.04	0.12	2.68	100.06	4.88
dA	y = 295.259 + 5284.18x, 0.9997	0.12~11.50	0.04	0.12	1.50	2.99	0.99	3.32	98.5	3.29
dC	y = 157.122 + 4241.14x, 0.9971	0.09~8.80	0.03	0.09	3.70	4.29	0.23	3.40	96.9	3.98
dG	y = 1235.86 + 44695.6x, 1.0000	0.09~8.77	0.01	0.03	2.87	3.28	0.42	2.88	97.7	3.74
dU	y = − 13.5999 + 1756.23x, 0.9988	0.09~9.16	0.03	0.09	2.48	3.58	0.37	3.95	100.12	4.05
AMP	y = 440.578 + 8906.34x, 0.9989	0.10~10.40	0.01	0.03	3.25	3.97	0.75	3.45	97.7	4.46
CMP	y = − 264.393 + 5715.24x, 0.9998	0.10~9.88	0.01	0.03	3.56	4.42	2.74	1.65	99.6	3.59
GMP	y = 467.242 + 6476.87x, 0.9978	0.11~11.28	0.01	0.03	3.46	4.49	3.80	1.83	98.4	3.03
UMP	y = − 47.7751 + 7174.29x, 0.9996	0.12~11.48	0.01	0.03	2.64	2.92	0.41	2.36	95.8	4.20

**Table 3:** The contents of 15 nucleic acid constituents in 80 *T. matsutake* samples (mg/100 g).

No.	Location	A	C	G	I	T	U	X	dA	dC	dG	dU	AMP	CMP	GMP	UMP	Total
S-01	Jiulong	8.41	2.49	6.97	1.07	0.01	5.09		0.78	0.21	0.29		0.87	2.32	3.06	0.52	32.08 ± 2.73
S-02	Jiulong	8.11	2.45	7.41	1.16	0.24	5.47		0.60	0.14	0.32	0.06	0.88	2.31	3.24	0.47	32.87 ± 2.75
S-03	Jiulong	12.83	3.40	10.90	1.24	0.15	7.56		0.69		0.34		0.88	3.16	4.69	0.53	46.39 ± 4.35
S-04	Jiulong	15.22	3.56	11.32	1.26	0.03	7.61		0.86		0.38	0.24	0.86	3.22	4.62	0.50	49.67 ± 4.79
S-05	Jiulong	12.29	3.48	11.68	1.54	0.20	7.30	0.12	0.99	0.23	0.42	0.37	0.75	2.74	3.80	0.41	46.33 ± 4.11
S-06	Jiulong	9.11	2.71	7.92	0.91		5.56		0.58	0.14	0.32	0.29	0.67	1.15	1.61	0.29	31.25 ± 3.09
S-07	Jiulong	7.89	1.93	6.10	0.94		4.70		0.37	0.02	0.19		0.95	2.31	2.74	0.58	28.71 ± 2.55
S-08	Jiulong	14.53	3.28	11.02	1.38		7.67		0.61	0.05	0.32	0.33	0.87	2.54	3.87	0.40	46.87 ± 4.64
S-09	Jiulong	14.19	3.03	11.56	1.27		7.29		0.75	0.09	0.39	0.26	0.90	3.94	5.24	0.53	49.43 ± 4.62
S-10	Jiulong	10.44	2.97	8.17	1.15		6.09		0.87	0.29	0.43	0.22	1.07	2.92	4.06	0.54	39.22 ± 3.32
S-11	Jiulong	12.00	2.83	9.38	1.06	0.49	6.66		0.92	0.08	0.36	0.20	0.46	1.69	2.96	0.39	39.46 ± 3.80
S-12	Jiulong	13.09	2.88	10.00	1.23		6.69		0.71	0.05	0.31	0.11	1.97	4.85	6.62	0.58	49.09 ± 4.21
S-13	Jiulong	7.84	1.90	6.61	0.86		4.35		0.47	0.00	0.24	0.10	1.62	2.88	3.24	0.53	30.65 ± 2.55
S-14	Jiulong	13.66	3.12	10.77	1.13	0.37	5.83		0.77		0.28	0.10	1.32	3.82	5.94	0.51	47.62 ± 4.34
S-15	Jiulong	14.70	2.50	10.99	1.11		6.88		1.01	0.17	0.34		1.72	5.67	8.16	0.72	53.99 ± 4.79
S-16	Jiulong	10.64	2.73	9.31	1.22	0.67	5.92		0.94		0.49	0.15	0.82	2.43	3.41	0.45	39.17 ± 3.49
S-17	Jiulong	14.93	3.32	12.35	1.58	0.16	8.24	0.03	0.91	0.17	0.41	0.15	1.12	3.66	4.88	0.48	52.38 ± 4.73
S-18	Jiulong	12.40	2.57	9.52	1.11	0.37	6.24		0.77		0.43		0.75	2.42	3.35	0.47	40.40 ± 3.98
S-19	Jiulong	10.72	2.58	9.85	0.85		6.93		0.80		0.40	0.19	1.20	2.87	3.53	0.41	40.34 ± 3.75
S-20	Jiulong	5.58	1.09	3.56	0.48	0.03	3.12		0.20		0.09		0.49	1.07	1.49	0.48	17.68 ± 1.73
S-21	Jiulong	10.71	2.33	9.31	1.15	0.37	6.04		0.70		0.30	0.10	1.04	2.81	4.42	0.55	39.82 ± 3.56
S-22	Jiulong	11.06	2.54	8.82	1.06		6.13		1.09	0.21	0.47		0.94	2.78	3.29	0.48	38.87 ± 3.57
S-23	Jiulong	15.45	2.97	11.81	1.17	0.21	7.98	0.01	1.21		0.38		1.50	6.67	8.79	0.72	58.88 ± 5.10
S-24	Jiulong	11.68	2.81	9.08	1.04		6.10		0.95	0.10	0.33	0.17	1.18	3.95	5.94	0.62	43.96 ± 3.77
S-25	Jiulong	11.92	3.76	11.02	1.14	0.44	7.40		0.89	0.21	0.42	0.05	0.23	1.39	2.06	0.36	41.30 ± 4.11
S-26	Jiulong	14.04	2.82	10.35	1.32		7.14		0.64		0.33	0.30	1.64	5.45	8.18	0.70	52.92 ± 4.59
S-27	Jiulong	12.63	2.11	8.23	1.12		5.49	0.19	0.84	0.08	0.32		2.18	8.02	8.97	0.94	51.12 ± 4.22
S-28	Jiulong	14.93	3.37	11.71	1.37	0.40	7.87	0.00	1.18	0.18	0.48	0.29		5.23	6.38	0.62	54.04 ± 4.78
S-29	Jiulong	10.35	3.53	6.95	1.61	0.49	6.23	0.05	1.42	0.12	0.48	0.35	2.70	5.36	6.59	0.66	46.90 ± 3.22
S-30	Jiulong	8.55	2.87	6.22	1.49	0.19	7.48	0.06	0.82		0.37	0.36	2.70	3.86	5.41	0.77	41.15 ± 2.91
S-31	Xiaojin	9.30	2.19	6.76	1.28		7.64		0.44	0.03	0.28		0.71	1.74	2.82	0.26	33.44 ± 3.24
S-32	Xiaojin	9.55	2.24	7.52	1.62	0.13	8.27	0.00	0.47		0.19	0.24	0.79	1.66	2.37	0.16	35.22 ± 3.33
S-33	Xiaojin	6.52	1.25	5.90	1.21		5.52	0.04	0.62		0.33		0.75	1.48	2.47	0.28	26.37 ± 2.38
S-34	Xiaojin	9.40	2.27	7.71	1.82		8.49	0.08	0.44	0.08	0.32		0.74	1.68	2.44	0.21	35.69 ± 3.42
S-35	Xiaojin	10.06	2.20	8.31	2.21		11.26	0.03	0.30	0.10	0.35	0.14	0.77	1.61	2.51	0.19	40.03 ± 3.94
S-36	Xiaojin	8.95	2.15	7.18	1.66	0.28	8.45	0.05	0.46	0.09	0.28		0.64	0.96	1.71	0.15	33.01 ± 3.25
S-37	Xiaojin	9.20	2.04	7.22	1.84	0.38	10.02	0.06	0.44		0.25	0.11	0.47	1.05	1.71	0.12	34.93 ± 3.53
S-38	Xiaojin	9.51	2.32	7.14	1.83	0.17	7.96	0.02	0.34		0.21		0.75	1.45	2.25	0.18	34.13 ± 3.31
S-39	Xiaojin	7.79	2.11	6.04	1.05	0.27	6.66		0.41	0.10	0.24			0.65	0.84	0.13	26.30 ± 2.88
S-40	Xiaojin	7.48	1.73	5.71	1.07	0.23	6.41		0.25	0.04	0.18	0.03	0.35	0.53	0.80	0.11	24.91 ± 2.64
S-41	Xiaojin	7.92	2.02	6.72	1.64	0.26	6.98		0.22	0.06	0.20		0.64	1.28	1.80	0.19	29.94 ± 2.89
S-42	Xiaojin	6.48	2.04	5.67	1.45	0.41	6.09		0.50		0.32	0.05		0.68	0.89	0.14	24.72 ± 2.49
S-43	Xiaojin	10.13	2.14	8.16	1.76	0.25	9.41		0.38	0.08	0.26		0.77	1.58	2.44	0.16	37.52 ± 3.72
S-44	Xiaojin	9.54	1.87	7.14	1.49	0.24	8.51	0.12	0.50	0.10	0.33		1.53	2.62	4.11	0.31	38.41 ± 3.30
S-45	Xiaojin	8.80	1.99	5.93	1.23		7.44	0.08	0.33	0.02	0.17	0.07	0.40	1.06	1.71	0.18	29.41 ± 2.99
S-46	Xiaojin	8.99	2.05	7.43	1.49	0.39	8.33	0.01	0.47	0.13	0.21	0.14	0.96	1.83	2.60	0.22	35.26 ± 3.17
S-47	Xiaojin	8.71	2.22	6.82	1.46	0.59	7.12		0.30		0.21	0.08	0.59	1.28	2.04	0.20	31.61 ± 3.03
S-48	Xiaojin	10.60	2.40	7.67	1.76	0.28	9.31	0.02	0.37	0.15	0.26	0.10	0.61	1.51	2.80	0.18	38.02 ± 3.60
S-49	Xiaojin	9.17	1.83	7.13	2.11	0.46	8.24		0.38	0.08	0.31	0.23	1.08	1.78	2.73	0.23	35.76 ± 3.18
S-50	Xiaojin	8.72	2.11	7.22	1.41	0.35	7.26		0.64	0.09	0.45	0.29	0.57	1.53	2.24	0.21	33.10 ± 3.01
S-51	Xiaojin	7.93	1.79	6.37	1.31	0.34	7.78		0.33	0.10	0.26	0.31	1.00	1.55	2.33	0.21	31.60 ± 2.87
S-52	Xiaojin	9.73	2.02	6.90	1.89	0.23	9.44	0.07	0.43	0.07	0.31	0.09	1.33	2.36	3.46	0.28	38.60 ± 3.37
S-53	Xiaojin	8.80	1.96	5.95	1.71	0.21	7.79	0.01	0.38	0.03	0.16	0.15	0.41	0.89	1.42	0.22	30.09 ± 2.97
S-54	Xiaojin	7.80	1.89	6.73	2.13	0.08	8.20	0.04	0.25	0.02	0.21	0.15	0.66	1.43	1.75	0.19	31.52 ± 2.94
S-55	Xiaojin	6.92	1.95	6.17	1.04	0.17	6.33		0.49		0.33	0.08	0.72	1.17	1.91	0.24	27.52 ± 2.56
S-56	Xiaojin	8.44	1.96	6.63	1.27	0.45	7.73	0.03	0.45	0.14	0.24	0.21	0.58	1.18	2.11	0.19	31.63 ± 2.93
S-57	Xiaojin	7.17	1.86	6.33	1.62	0.50	5.89		0.26	0.11	0.21	0.26	0.72	1.10	1.53	0.19	27.74 ± 2.51
S-58	Xiaojin	7.94	1.62	6.10	1.47	0.15	8.19		0.27		0.18	0.07	0.66	1.32	1.64	0.16	29.75 ± 3.01
S-59	Xiaojin	10.36	2.36	8.51	2.06		9.45	0.11	0.42	0.03	0.36	0.17	0.99	2.31	3.65	0.23	41.01 ± 3.71
S-60	Lixian	8.41	2.49	6.97	1.07	0.01	5.09		0.78	0.21	0.29		0.87	2.32	3.06	0.52	32.09 ± 2.74
S-61	Lixian	8.11	2.45	7.41	1.16	0.24	5.47		0.6	0.14	0.32	0.06	0.88	2.31	3.24	0.47	32.86 ± 2.75
S-62	Lixian	8.24	2.16	6.87	1.09	0.15	6.87		0.54	0.11	0.30	0.03	0.73	2.14	2.97	0.25	32.45 ± 2.87
S-63	Lixian	9.34	2.38	8.57	1.26	0.22	6.51	0.02	0.41	0.16	0.33	0.11	0.81	1.92	3.87	0.19	36.10 ± 3.20
S-64	Lixian	8.15	2.61	7.12	1.18	0.15	5.95	0.01	0.32	0.07	0.19	0.09	0.67	1.59	2.18	0.16	30.44 ± 2.76
S-65	Lixian	9.42	2.34	6.93	1.02	0.24	6.81	0.04	0.27	0.05	0.26	0.10	0.89	1.85	2.63	0.21	33.06 ± 3.03
S-66	Muli	12.83	3.4	10.9	1.24	0.15	7.56		0.69		0.34		0.88	3.16	4.69	0.53	46.37 ± 4.35
S-67	Muli	15.22	3.56	11.32	1.26	0.03	7.61		0.86		0.38	0.24	0.86	3.22	4.62	0.5	49.68 ± 4.79
S-68	Muli	12.64	2.14	9.76	1.01	0.08	8.29	0.02	0.35	0.03	0.39	0.33	0.91	2.37	3.34	0.36	42.02 ± 4.05
S-69	Muli	13.95	2.17	8.79	1.76	0.17	9.73		0.37		0.31	0.05	0.72	2.51	5.31	0.18	46.02 ± 4.53
S-70	Kangding	9.3	2.19	6.76	1.28		7.64		0.44	0.03	0.28		0.71	1.74	2.82	0.26	33.45 ± 3.24
S-71	Kangding	9.55	2.24	7.52	1.62	0.13	8.27		0.47	0.06	0.27	0.24	0.79	1.66	2.37	0.16	35.35 ± 3.33
S-72	Kangding	10.34	1.99	6.93	1.37	0.3	9.78	0.07	0.54	0.15	0.33	0.18	0.93	1.46	3.57	0.23	38.17 ± 3.54
S-73	Kangding	7.99	2.07	8.25	1.82	0.24	8.73	0.02	0.49	0.11	0.24	0.36	0.85	2.87	2.28	0.19	36.51 ± 3.18
S-74	Kangding	8.34	1.68	7.61	1.46	0.26	8.26		0.32		0.37	0.27	0.67	1.98	2.72	0.19	34.13 ± 3.20
S-75	Luding	9.54	2.11	8.38	2.21	0.15	7.96		0.44	0.09	0.21	0.15	0.82	2.74	1.78	0.85	37.43 ± 3.35
S-76	Luding	8.83	2.17	8.18	1.84	0.28	6.67	0.07	0.54	0.1	0.19	0.27	0.77	2.93	1.9	0.34	35.08 ± 3.03
S-77	Luding	8.14	2.53	9.34	1.49	0.07	8.29	0.06	0.47	0.14	0.31	0.33	0.59	2.35	2.34	0.56	37.01 ± 3.29
S-78	Yajiang	8.92	1.97	8.16	1.05	0.21	8.31		0.39		0.18	0.09	0.83	1.67	1.1	0.66	33.54 ± 3.40
S-79	Yajiang	9.37	2.28	7.38	1.83	0.02	9.27	0.03	0.5	0.08	0.3	0.29	0.91	1.92	3.65	0.48	38.31 ± 3.35
S-80	Yajiang	8.16	2.54	7.91	1.17	0.19	8.99	0.02	0.51	0.05	0.28	0.24	0.56	1.85	2.91	0.53	35.91 ± 3.22
		10.08 ± 2.40	2.40 ± 0.55	8.11 ± 1.84	1.38 ± 0.35	0.25 ± 0.15	7.32 ± 1.42	0.05 ± 0.04	0.58 ± 0.26	0.10 ± 0.06	0.30 ± 0.08	0.18 ± 0.10	0.92 ± 0.45	2.39 ± 1.38	3.34 ± 1.80	0.37 ± 0.20	

## Supplementary Materials

The following are available online.
