# Supplementary material for: Quality Evaluation of Tricholoma matsutake Based on the Nucleic Acid Compounds by UPLC-TOF/MS and UPLC-QqQ/MS
Source: Molecules. 2018 Dec 21;24(1):34. doi: 10.3390/molecules24010034 (PMC6337655; doi:10.3390/molecules24010034)
Supplement: Supplementary file 1 [file molecules-24-00034-s001.pdf]

Table S1 Comparison of analytical performances reported for determination of nucleic acid constituents

Analyte	UPLC/MS [this work]	HPLC/UV [Ref S1]	HPLC/MS [Ref S2]	UPLC/MS [Ref S3]
A	0.12~11.15 ng/L*, 0.04 ng[#], 0.12 ng[&], 99.1%[δ]	0.02~9.99 μg/mL, 0.003 μg/mL, 0.010 μg/mL, 77.3%	3.82~4890 ng/mL, 0.29 ng/mL, 0.96 ng/mL, 89.62%	0.002~1.152 μg/mL, 0.11 ng/mL, 0.29 ng/mL, 97.2%
C	0.10~9.95 ng/L, 0.01 ng, 0.03 ng, 97.2%	0.08~33.2 μg/mL, 0.020 μg/mL, 0.070 μg/mL, 71.0%	41.90~26800 ng/mL, 1.57 ng/mL, 5.24 ng/mL, 93.54%	0.005~1.144 μg/mL, 0.57 ng/mL, 2.28 ng/mL, 93.1%
G	0.10~9.87 ng/L, 0.01 ng, 0.13 ng, 98.5%	0.03~11.9 μg/mL, 0.003 μg/mL, 0.010 μg/mL, 96.6%	5.58~2860 ng/mL, 0.11 ng/mL, 0.35 ng/mL, 104.28%	0.003~1.474μg/mL, 0.12 ng/mL, 0.37 ng/mL, 103.1%
I	0.12~11.68 ng/L, 0.01 ng, 0.13 ng, 98.9%		10.80~692 ng/mL, 0.81 ng/mL, 2.70 ng/mL, 92.56%	0.005~2.256 μg/mL, 0.14 ng/mL, 0.56 ng/mL, 97.9%
T	0.10~9.92 ng/L, 0.03 ng, 0.09 ng, 96.3%		5.20~166 ng/mL, 0.39 ng/mL, 1.30 ng/mL, 112.69%	0.010~3.936 μg/mL, 0.66 ng/mL, 1.97 ng/mL, 93.4%
U	0.09~8.91 ng/L, 0.03 ng, 0.09 ng, 98.6%	0.03~10.8μg/mL, 0.006 μg/mL, 0.020 μg/mL, 92.7%	10.50~2690 ng/mL, 0.19 ng/mL, 0.66 ng/mL, 96.73%	0.017~3.376 μg/mL, 0.84 ng/mL, 2.52 ng/mL, 91.6%
X	0.10~9.83 ng/L, 0.01 ng, 0.13 ng, 100.06%			
dA	0.12~11.50 ng/L, 0.04 ng, 0.12 ng, 98.5%		10.50~335 ng/mL, 0.79 ng/mL, 2.63 ng/mL, 83.56%	0.002~1.220 μg/mL, 0.12 ng/mL, 0.31 ng/mL, 99.1%
dC	0.09~8.80 ng/L, 0.03 ng, 0.09 ng, 96.9%		1.30~334 ng/mL, 0.19 ng/mL, 0.65 ng/mL, 95.43%	0.006~2.925 μg/mL, 0.59 ng/mL, 2.36 ng/mL, 95.6%
dG	0.09~8.77 ng/L, 0.01 ng, 0.13 ng 97.7%		10.30~2640 ng/mL, 1.55 ng/mL, 5.15 ng/mL, 90.78%	0.003~1.280 μg/mL, 0.31 ng/mL, 0.64 ng/mL, 100.5%
dU	0.09~9.16 ng/L, 0.03 ng, 0.09 ng, 100.12%		5.22~167 ng/mL, 0.78 ng/mL, 2.61 ng/mL, 107.21%	0.014~2.740 μg/mL, 1.70 ng/mL, 6.85 ng/mL, 96.4%
AMP	0.10~10.40 ng/L, 0.01 ng, 0.13 ng, 97.7%	0.03~10.8 μg/mL, 0.006 μg/mL, 0.020 μg/mL, 87.0%		
CMP	0.10~9.88 ng/L, 0.01 ng, 0.13 ng, 99.6%	0.10~40.0 μg/mL, 0.014 μg/mL, 0.050 μg/mL, 84.9%		0.020~3.900 μg/mL, 2.44 ng/mL, 9.75 ng/mL, 94.7%
GMP	0.11~11.28 ng/L, 0.01 ng, 0.13 ng, 98.4%	0.10~40.9 μg/mL, 0.009 μg/mL, 0.030 μg/mL, 68.6%		
UMP	0.12~11.48 ng/L, 0.01 ng, 0.13 ng, 95.8%	0.10~41.4 μg/mL, 0.014 μg/mL, 0.050 μg/mL, 98.2%		

*linear range, [#]LOD, [&]LOD, [δ]recovery

[Ref S1] Ranogajec, A., S. Beluhan, and Z. Smit, Analysis of nucleosides and monophosphate nucleotides from mushrooms with reversed-phase HPLC. Journal of Separation Science, 2010. 33(8): 1024-1033.

[Ref S2] Wang, H., et al., Simultaneous Determination of 16 Nucleosides and Nucleobases in Euryale ferox Salisb. by Liquid Chromatography Coupled with Electro Spray Ionization Tandem Triple Quadrupole Mass Spectrometry (HPLC-ESI-TQ-MS/MS) in Multiple Reaction Monitoring (MRM) Mode. Journal of Chromatographic Science, 2015. 53(8): p. 1386-1394.

[Ref S3] Guo, S., et al., Hydrophilic interaction ultra-high performance liquid chromatography coupled with triple quadrupole mass spectrometry for determination of nucleotides, nucleosides and nucleobases in Ziziphus plants. Journal of Chromatography A, 2013. 1301: p. 147-155.
